# 

**Published:** 1890-09-27

**Authors:** 


**An Endless Quest** - 385

**Words of Consolation.**—God's Ministers - 386

**The Doctor's Pulpit.**—Commonplace - 387

**Working Women in Large Towns.**—XI.—Shop Assistants - 388

**Sympathetic Surgery.**—II. - 389

**The History and Capabilities of Herbal Simples.**—XIX.—The Horse Chestnut - 390

**Everybody's Page** - 391

**Notes and News** - 392

**The Story of the Insane from Year to Year** - 393

**Zenana Bible and Medical Mission** - 399

**Annotations.**—Boy Suicides and Doctors—No Gentleman Keeps a Bull-dog—Cardinal Livagerie and Man Hunting - 394

**The Practitioner's Mirror and Retrospect**:—

Surgery—Abscess of Bone—The Treatment of Cysts, 395; Optic Neuritis, 396; Vesical Tumours—Guinea-Worm, 397; Carcinoma of the Larynx - 398

New Drugs, Appliances, and Things Medical—"Bontha" - 398

**Editor's Letter-box**—Out-patients in Hospitals - 39[illegible]

**Students' Column** - 399

**Passing Topics.**—A Canterbury Tale - 400

**General Charities** - 400

### EXTRA SUPPLEMENT—THE NURSING MIRROR.

**En Passant.**—Dartmouth Cottage Hospital—New Medical School for Women—Notes and Queries—The Rural Nursing Association—In Russia—The Dufferin Fund—Miss Kate Marsden—Hints for Christmas—Our Duty - cvii

**Sketches of Insanity.** By Francis H. Walmsley, M.D.—XII.—Puerperal Insanity - cviii

**A Catholic Nurse's Holiday** - cviii

**Six Months in a Hospital.** By a "Special Pro." - cix

**Appointments** - cx

**Notes and Queries** - cx

**Vacancies** - cx

**Amusements and Relaxation** - cx

